# CYP21A2 Intron 2 Genetic Variants Might Be Associated with the Clinical Characteristics of Women with PCOS

**DOI:** 10.3390/biomedicines12071528

**Published:** 2024-07-09

**Authors:** Ralitsa Robeva, Silvia Andonova, Tihomir Todorov, Aylin Feyzullova, Atanaska Elenkova, Georgi Kirilov, Alexey Savov, Sabina Zacharieva, Albena Todorova

**Affiliations:** 1 Department of Endocrinology, Medical Faculty, Medical University-Sofia, USHATE “Acad. Iv. Penchev”, 1000 Sofia, Bulgariaatanaskae@gmail.com (A.E.);; 2 National Genetic Laboratory, Medical Faculty, Medical University-Sofia, University Hospital of Obstetrics and Gynecology “Maichin Dom”, 1000 Sofia, Bulgaria; sandonova@netscape.net (S.A.);; 3 Genetic Medico-Diagnostic Laboratory “Genica”, 1000 Sofia, Bulgaria; 4 Department of Medical Chemistry and Biochemistry, Medical Faculty, Medical University-Sofia, 1000 Sofia, Bulgaria

**Keywords:** PCOS, non-classic CAH, IVS2-13A/C>G, rs6467, rs6453, rs6451, rs369651496, rs6474

## Abstract

Aims: Pathogenic variants in the *CYP21A2* gene are related to the classic and non-classic forms of congenital adrenal hyperplasia (CAH). However, the role of CAH carrier status in the clinical presentation of polycystic ovarian syndrome (PCOS) is still unclear. Moreover, the possible associations of different *CYP21A2* gene polymorphisms with metabolic and reproductive abnormalities in PCOS have not been investigated. Therefore, the present study aims to examine the prevalence of the most common *CYP21A2* pathogenic variant IVS2-13A/C>G (c.293-13A/C>G) in Eastern European women with PCOS and to evaluate the associations between common intron 2 genetic polymorphisms and the clinical symptoms of the patients. Methods: Sixty consecutively recruited women with PCOS were genotyped for the *CYP21A2* intron 2 IVS2-13A/C>G genetic variant. Additionally, *CYP21A2* intron 2 polymorphic variants rs6453 (c.293-44G>T), rs6451 (c.293-67C>A/G), rs369651496 (c.293-104del), and rs6474 (c.308G>A/p.R103L) were tested and described. The clinical and hormonal characteristics were compared in women with PCOS and with polymorphic and wild-type genotypes. Results: The heterozygous *CYP21A2* pathogenic variant IVS2-13A/C>G was found in one of the investigated PCOS patients (1.67%) with a non-hyperandrogenic type of PCOS. The presence of the rs6453 (c.293-44G>T) T-allele was associated with increased levels of DHEAS (15.18 vs. 9.14 µmol/L, *p* = 0.003) compared to the wild-type genotype in the investigated group. The rs6451 (c.293-67C>A/G) minor alleles were associated with an earlier age of menarche in the patients (12.0 vs. 13.0 years, *p* = 0.007). The polymorphic rs369651496 minor 6G allele was related to a better lipid profile in the women with PCOS, while the rs6474 variant modulated the blood pressure of the patients. Conclusions: The presence of *CYP21A2* genetic minor alleles of rs6467 (IVS2-13A/C, c.293-13A/C), rs6453 (c.293-44G>T), rs6451 (c.293-67C>A/G), rs369651496 (c.293-104del), and rs6474 (c.308G>A/p.R103L) might modulate the adrenal androgens, age of menarche, and metabolic features in women with PCOS. Further studies on 21-hydroxylase genetic variants (pathogenic and polymorphisms) in different ethnic groups might help reveal the influence of adrenal steroidogenesis on PCOS development, clinical manifestations, and lifelong cardiovascular risks.

## 1. Introduction

Cytochrome 450 21-hydroxylase (CYP21A2) is a microsomal monooxygenase expressed in the adrenal cortex, which is involved in the synthesis pathways of cortisol, aldosterone, and androgens [1]. It catalyzes the conversion of progesterone and 17-OH-progesterone to deoxycorticosterone and 11-deoxycortisol, respectively [2]. Pathogenic variants in the *CYP21A2* gene might lead to impaired enzyme activity, disturbances in mineral- and glucocorticoid production, and chronic overstimulation of the hypothalamus–pituitary–adrenal axis, resulting in an abundance of androgen precursors [3]. The degree of enzyme deficiency depends on the type and position of the genetic variant and determines the clinical characteristics of patients with congenital adrenal hyperplasia (CAH) [3,4]. An enzyme function decline below 1–2% causes classical salt-wasting or simple-virilizing CAH forms, with significant impairment of cortisol and aldosterone production and severe hyperandrogenism; conversely, mild disturbances of enzyme activity result in the non-classical CAH form (NC CAH), characterized mainly by symptomatic or asymptomatic hyperandrogenism [5,6].

The worldwide prevalence of NC CAH has been estimated to be 4.2% among women with hyperandrogenic symptoms, so its exclusion is essential for the proper diagnosis of other hyperandrogenic states, e.g., polycystic ovarian syndrome (PCOS) [7,8]. However, the prevalence of *CYP21A2* heterozygous carriers is not estimated adequately among women with PCOS, though it is reported to be relatively high in the general population (1:60 individuals) [9]. Currently, the significance of CAH carrier status for the development of PCOS is unclear, but a recent Italian study has shown hyperandrogenic symptoms in more than 80% of CAH heterozygous women [10]. Thus, further studies are needed to reveal the possible role of the presence of heterozygous *CYP21A2* genetic variants in the clinical features of PCOS. Additionally, the investigation of common *CYP21A2* polymorphisms among PCOS women might be of clinical importance, considering their possible effects on adrenal androgen synthesis.

Ethnicity is considered a key factor affecting the prevalence of heterozygous carriers of CAH. For instance, in Central European populations, the heterozygosity for mild and severe *CYP21A2* mutations is much higher than expected, reaching 9.5% of clinically healthy individuals [11]. The most common severe *CYP21A2* genetic variant among CAH patients is IVS2-13A/C>G (c.293-13A/C>G), with an allelic frequency of 20–30% among European and American CAH individuals [12]. However, in Central and Eastern European countries, the prevalence of the same variant is significantly higher, affecting 40% to 60% of CAH patients [13,14,15,16]. On the other hand, the IVS2-13A/C>G variant has been found in a heterozygous state in only one of 400 healthy individuals from the same geographical area [11]. Thus, it would be intriguing to assess the frequency of this genetic variant among PCOS patients and whether it is associated with significant hyperandrogenic symptoms in the affected women.

Therefore, the present study aims to investigate the prevalence of the IVS2-13A/C>G (c. 293-13A/C>G) variant in the population of Bulgarian (Eastern European) women with PCOS. Moreover, it aims to reveal the possible clinical interrelations between the common polymorphisms located in the *CYP21A2* intron 2 and the clinical manifestations of PCOS, which have not been explored before. 

## 2. Methods

### 2.1. Participants and Laboratory Measurements

Sixty patients (median age 23.50 (18–45) years) with PCOS who were consulted in the Department of Endocrinology from August 2023 to January 2024 were recruited consecutively. All of them complained of hirsutism, acne, menstrual irregularities, obesity, and/or infertility and fulfilled the ESHRE criteria for PCOS [8]. Patients with severe concomitant diseases, liver or kidney insufficiency, psychiatric diseases, or pregnancy were not included in the study. Two of the patients had received oral contraceptives in the previous three months, and 21 of the women were on metformin treatment. In all patients, the presence of congenital adrenal hyperplasia, Cushing syndrome, prolactinoma, and other causes for hyperandrogenism and menstrual disturbances were excluded by the appropriate hormonal tests. Hypothyroidism had been diagnosed in 10 patients who were euthyroid on L-thyroxin treatment. All patients underwent a complete physical assessment, including blood pressure measurement, and anthropometric data collection, including height, weight, and body mass index (BMI) calculation. The presence of hirsutism and acne was also registered. The age of menarche and the presence of live births were self-reported. In all patients, the levels of fasting glucose, high-density lipoprotein cholesterol levels (HDL-ch), low-density lipoprotein cholesterol levels (LDL-ch), triglycerides (TG), and total cholesterol were measured enzymatically by an automatic analyzer (Roche Cobas E411). Additionally, total testosterone (T), dehydroepiandrosterone-sulfate (DHEAS), immunoreactive insulin (IRI), luteinizing hormone (LH), follicle-stimulating hormone (FSH), thyroid-stimulating hormone (TSH), and prolactin were determined by еlectrochemiluminiscence methods (Roche Cobas E411). The intra-assay coefficients were below 3.2%, while inter-assay coefficients were below 5.3% for all parameters. The androstenedione and 17-OH progesterone (17OHPg) were measured by the radioimmunoassay (RIA) method (Diasource, Belgium) with intra-assay coefficients below 4.6% and inter-assay coefficients below 7.6%.

An oral glucose tolerance test was performed on 50 patients. Four patients had impaired fasting glucose, three had impaired glucose tolerance, and two had diabetes mellitus type 2. Hormonal investigations were performed in the early follicular phase of amenorrhea. K_2_EDTA venous blood samples for genetic tests were also collected. The experimental protocol was explained to all participants, and written informed consent was obtained. The study was approved by the Ethics Committee of the Medical University—Sofia (Protocol №09/23.06.2023).

### 2.2. Genetic Investigations

DNA from all participants was obtained by a standard salt-extraction method. Because the *CYP21A2* gene and the non-processed pseudogene *CYP21A1P* share up to 98% and 96% sequence homology in exons and non-coding sequences, respectively [17], genetic analysis was performed with the use of highly specific primers according to a cascade strategy [18]. In order to investigate the frequency of the IVS2-13A/C>G genetic variant, an amplification of the target region was performed; the initially generated PCR product according to the in-house modified protocol was subsequently sequenced with specific internal primers (available on request). Amplifications were performed in a final 25 μL-reaction volume which contained 1 μg of genomic DNA, 0.5 μM for each primer, 0.5 mM of each deoxynucleotide triphosphate, and 1 U of *Taq* DNA polymerase with the appropriate Buffer with 2.0 mM of MgCl_2_ (GenetBio, Daejeon, Republic of Korea). The following thermocycling conditions were used for the amplification: 5 min at 94 °C (initial denaturation), followed by 35 cycles of denaturation (30 s 94 °C), annealing (40 s at 60 °C), and extension (1:30 min at 72 °C), with a final extension of 10 min at 72 °C. The region of interest was then scanned by a Sanger sequencing analysis using a BigDyeTerminator Cycle Sequencing Kit, v.3.1 (Thermo Fisher Scientific, Waltham, MA, USA) according to the manufacturer’s protocol. An ABI 3130 Genetic DNA analyzer (Thermo Fisher Scientific, USA) was used for the identification of the genetic variants in the target region of the gene. *CYP21A2* NM_000500.9 was used as a reference sequence; a visualization of the electropherograms was made using the SeqScape software v2.7 (Thermo Fisher Scientific, USA).

### 2.3. Statistical Analysis

The results were presented as a median with an interquartile range for continuous variables or as a frequency (%). Chi-square and Fisher’s exact tests were used to explore differences between categorical variables. After a Kolmogorov–Smirnov test was performed to determine the normality of the distribution, a Mann–Whitney U test was used to evaluate the differences between the two groups. A *p*-level < 0.05 was considered statistically significant. The data were analyzed by MedCalc^®^ Statistical Software version 22.021 (MedCalc Software Ltd., Ostend, Belgium; https://www.medcalc.org (accessed on 1 May 2024)). 

## 3. Results

**Figure 1:**
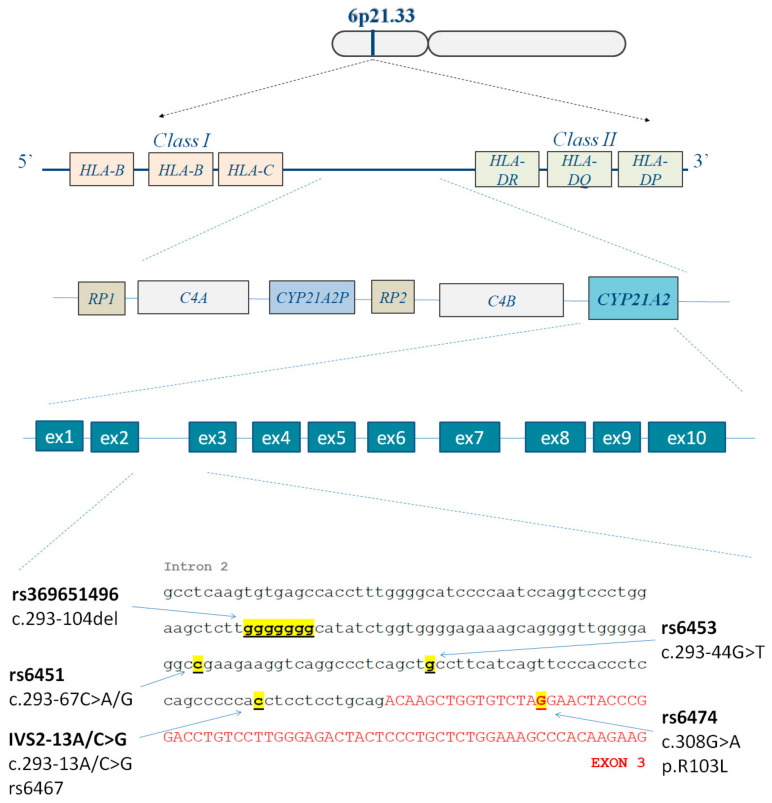
Illustration of the positions of the investigated *CYP21A2* variants IVS2-13A/C>G (rs6467, c.293-13A/C>G), rs6453 (c.293-44G>T), rs6451 (c.293-67C>A/G), rs369651496 (c.293-104del), rs6474 (c.308G>A/p.R103L), and the localization of the gene (short arm of chromosome 6).

The clinical and laboratory characteristics of the investigated women are presented in Table 1. The most common phenotype in the investigated women was phenotype A [hyperandrogenicity (HA) + chronic anovulation (CA) + polycystic ovaries (PCO)] − 63.3% (n = 38), while phenotype B (HA + CA without PCO) was present in 13.3% of women (n = 8). “Ovulatory” PCOS (HA + PCO) was observed in seven patients (11.7%). Additionally, seven women (11.7%) were with non-HA phenotype D (CA + PCO). The genetic investigation for the *CYP21A2* variant IVS2-13A/C>G (Figure 1) revealed one patient who was a heterozygous carrier (1.67% of all); the MLPA testing performed as a control analysis (MRC Holland, Amsterdam, The Netherlands) did not show any deletions/duplications in her DNA sample. She presented with normal weight, anovulation, polycystic ovaries, and an increased LH to FSH ratio, but no clinical or laboratory signs of hyperandrogenism (phenotype D) were observed and her basic 17-OH-progesterone levels were below 5 nmol/L.

### 3.1. IVS2-13A/C>G (c.293-13A/C>G), rs6467

The pathogenic variant IVS2-13A/C>G is located in intron 2 of the *CY21A2* gene and is characterized by a substitution of A or C nucleotide with G at 13 bp before the beginning of exon 3. The effect of this substitution is an abnormal splicing of intron 2 with the retention of 19 nucleotides in the mRNA and as a result of this, a shift in the translational reading frame [19,20]. The reported polymorphic C-allele frequency among Europeans is 0.56018, the polymorphic A-allele frequency is 0.43918, and the minor, pathogenic G-allele frequency is 0.00063 (https://www.ncbi.nlm.nih.gov/snp/rs6467 (accessed on 1 May 2024)). In our study, the clinical and laboratory characteristics of the women carrying the IVS2-13A/C>G genotype A/A (n = 24 [40.7%]) were compared to those in C/A carriers (n = 25 [42.4%]) and C/C carriers (n = 10 [16.9%])—Table 2. Interestingly, 20.8% of women with the IVS2-13 A/A genotype had at least one live birth compared to the 2.9% of other women (*p* = 0.036). The polymorphic allele C was not associated with the presence of acne, hirsutism, menstrual irregularities, or obesity. Women with the A/A genotype tended to show a lower diastolic blood pressure compared to C-allele heterozygous and homozygous carriers and they were with significantly lower insulin levels at the 120 min post glucose load (Table 3).

### 3.2. SNP rs6474 (c.308G>A, p.Arg103Lys)

This single nucleotide polymorphism has a reported minor allele A frequency of 0.31214 among Europeans (https://www.ncbi.nlm.nih.gov/snp/rs6474 (accessed on 1 May 2024)). In our group, the rs6474 G/G genotype was found in 30.0% (n = 18) of the patients, while the A/A genotype was observed in only 10.0% (n = 6) and the other 60.0% (n = 36) were heterozygous G/A carriers—Table 2. The prevalence of acne, hirsutism, obesity, and menstrual disturbances did not differ in women according to the rs6474 variants. However, rs6474 A-allele carriers had a higher systolic blood pressure and increased androstenedione levels compared to the patients with the G/G genotype (Table 4).

### 3.3. SNP rs6453 (c.293-44G>T)

The variant rs6453 c.293-44G>T has a major G-allele frequency among Europeans of 0.96551 and the frequency of the T-allele is 0.03449 (https://www.ncbi.nlm.nih.gov/snp/rs6453 (accessed on 1 May 2024)). The minor allele variant was observed in only six (10.0%) of the investigated PCOS patients in our study—Table 2. The presence of the polymorphic T-allele was associated with increased levels of DHEAS compared to the wild-type genotype (Table 5). The prevalence of acne, hirsutism, and menstrual disturbances did not differ in women with the polymorphic rs6453 T variant compared to the wild-type carriers, though obesity was not found among them (0% vs. 44%, *p* = 0.072).

### 3.4. SNP rs6451 (c.293-67C>A/G)

The frequency of the major allele C among Europeans is estimated to be 0.89067, allele A has a frequency of 0.07551, and allele G has a frequency of 0.02845 (https://www.ncbi.nlm.nih.gov/snp/rs6451 (accessed on 1 May 2024)). The investigation of rs6451 polymorphism in our study showed that the wild-type C/C genotype was present in 39 (65.0%) of the women with PCOS, while polymorphic alleles (A or G) were present in 21 (35.0%) of them—Table 2. Women with different rs6451 polymorphic variants had a similar prevalence of acne, hirsutism, obesity, and menstrual disturbances (Table 6). Women with variant A or G alleles showed an earlier age of menarche and decreased systolic blood pressure compared to C/C carriers (Table 6).

### 3.5. SNP rs369651496 (c.293-104del)

The presence of rs369651496 polymorphism was also investigated in the women with PCOS in our study. According to the data in dbSNP/NCBI, the European allele frequency of the GGGGGGG-allele (7G) is 0.86726, and frequency of the GGGGGG-allele (6G) is 0.13274 (https://www.ncbi.nlm.nih.gov/snp/rs369651496 (accessed on 1 May 2024)). In our study, more than half of the patients had the 7G/7G genotype (58.3%, n = 35), while 11.7% (n = 7) had the 6G/6G genotype and the other 30.0% (n = 18) were heterozygous 6G/7G carriers—Table 2. The rs369651496 7G/7G homozygous women had higher total cholesterol and LDL-cholesterol levels compared to other PCOS patients. No significant changes in other laboratory parameters were found (Table 7). Exclusion from the group of all treated patients (with oral contraceptives and metformin) did not change the results. 

## 4. Discussion

Our results demonstrated a frequency of 1.67% (1/60) of the *CYP21A2* genomic variant IVS2-13A/C>G in the group of consecutively recruited adult Eastern European PCOS patients diagnosed by the Rotterdam criteria. Notably, the identified heterozygous carrier was a lean woman with non-hyperandrogenic type PCOS. Thus, the selective screening of patients with hyperandrogenism only might underestimate the actual prevalence of CAH alleles in PCOS patients. In a German study of 21 PCOS patients, this heterozygous intron 2 splice site mutation was found in 3 of them (14.28%). Additionally, the pathogenic G-allele was observed in 3.6% (2/55) of adolescent Latvian PCOS patients, similar to the common population at the same age—2% (1/49) [21,22]. Witchel et al. found two IVS2-13 G-allele carriers among 109 American women with PCOS (1.83%), both with increased adrenal androgens—opposite to our patient [23]. The presence of the IVS2-13A/C>G variant has been described in 10% of Portuguese CAH carrier girls with clinical complaints suggesting hyperandrogenism, though non-symptomatic carriers have also been described among South European women [10,24]. However, this intron 2 splice mutation has not been found among *CYP21A2* genotyped 221 hirsute (PCOS or non-PCOS) patients and 252 healthy women from Denmark, emphasizing the critical influence of ethnicity on the genotype–phenotype correlations in PCOS [25]. 

Currently, distinguishing between PCOS and non-classic CAH is impossible based on clinical, basic hormonal, or metabolic data [26,27]. The morning levels of 17-OH progesterone are below the suggested cut-off in 13% of the non-classic CAH patients with a clarified genetic defect, but above it in 20–25% of PCOS women and in 7% of healthy individuals [28]. Therefore, the Synacthen test should be provided to identify non-classic CAH patients, but the same test has a low sensitivity for detecting heterozygous carriers [29,30]. The significance of heterozygous 21-hydroxylase gene mutations for clinical PCOS symptoms is still questionable, even in populations with a high prevalence of *CYP21A2* gene variants [20]. More extensive studies in diverse ethnic groups are needed to estimate the potential role of the specific 21-hydroxylase genetic variants for the pathogenesis, features, and reproductive outcome in PCOS patients with distinct phenotypes.

The influence of different SNPs in *CYP21A2* gene intron 2 on the hormonal and clinical characteristics in women with PCOS has not been investigated yet. The *CYP21A2* variants IVS2-13A/C (rs6467) and rs6474 G>A have been studied only among patients with autoimmune Addison disease (AAD) and acne [31,32]. The rs6467 and rs6474 heterozygous carriers of polymorphic alleles were found more frequently among patients with AAD than in non-affected controls [31]. The influence of both variants was dependent on HLA-loci, e.g., the IVS2-13A/C (rs6467) was in linkage disequilibrium with the well-known high-risk HLA-DRB1 haplotypes for autoimmune disturbances. Additionally, the rs6474 minor allele has been associated with severe acne in Chinese men, but not women [32].

The data from the investigated PCOS group in our study showed that IVS2-13A/C>G (rs6467) C-allele carrier status was associated with significantly increased insulin levels at 120 min post glucose load and a tendency for a higher diastolic blood pressure. Similarly, the rs6474 minor A-allele was related to a slightly increased systolic blood pressure as well as increased androstenedione levels. Additionally, the presence of the rs6451 minor allele (A or G) was also associated with differences in systolic blood pressure. The genetic variants included in our study were not associated with the presence of acne, hirsutism, menstrual irregularities, or obesity in the investigated women. The negative influence of the IVS2-13A/C (rs6467) C-allele and the rs6474 A-allele on blood pressure and hyperinsulinemia might be related to immunologic dysfunction considering the linkage equilibrium with HLA-loci and the close interrelations between chronic inflammation and vascular impairment [31,33]. The HLA-DRB1 genotype has been associated with increased autoantibody production against angiotensin AT_1_ receptors, which might cause predisposition to the development of hypertension [34]. On the other hand, polymorphisms might modulate adrenal steroid secretion, which could also affect the blood pressure of the patients [35]. The rs6474 G>A substitution leads to p.Arg103Lys change, located within 5Å of the substrate-binding site S1, critical for 21-hydroxylase function [32]. Slight differences in CYP21A2 activity might be associated with changes in androgens, glucocorticoid, and mineralocorticoid precursors leading to subtle blood pressure variations. However, measurements of cortisol, aldosterone, adrenocorticotropic hormone, renin, and 11-ketoandrogens have not been performed for the patients in our PCOS group, which is an important limitation of our study. Other significant limitations were the preselection of patients seeking endocrine help, which might not represent the common PCOS population, the relatively small number of genotyped patients, and the measurements of testosterone levels with the electrochemiluminiscent method instead of liquid chromatography/ tandem mass spectrometry. Further larger functional studies are needed in order to estimate the influence of different *CYP21A2* SNPs on enzyme kinetics and diverse steroid precursor production, as well as their causal interrelation with blood pressure and metabolic indices in women with PCOS.

It was interesting that both rs6451 (c.293-67C>A/G) A and G alleles were associated with an earlier age of menarche among the investigated women with PCOS. The age of menarche in adolescents with PCOS fluctuates significantly more than in non-affected women; therefore, PCOS patients with premature and delayed menarche have been described [36]. Menarche at an earlier age in some girls with PCOS might be related to different genetic and environmental factors, including obesity and genetic polymorphisms near the LIN28B locus [37]. Conversely, among untreated classic CAH patients, the age of menarche could be delayed because of pronounced hyperandrogenism [38]. However, the usual age of menarche in heterozygous CAH carriers is not well established and the possible role of *CYP21A2* SNPs for the pubertal development of healthy women, patients with PCOS, and non-classic CAH should be investigated further. The increased DHEAS levels in the variant rs6453 T-allele carriers showed that *CYP21A2* SNPs might be associated with variations in adrenal androgen production, but the clinical implications remain to be established.

The investigation of rs36951496 (c.293-104del) polymorphism in our group of women with PCOS showed a significant association with the lipid profile of the patients. The presence of the 6G-allele in the PCOS women was associated with lower total and LDL-cholesterol levels compared to the wild type carriers (7G/7G genotype). Further studies are needed to reveal if the same variant might influence cardiovascular risk in women with PCOS throughout their lifespan.

In conclusion, the pathogenic *CYP21A2* genetic variant IVS2-13A/C>G, which is the most common severe mutation for classical CAH, was found in 1.67% of the adult PCOS patients in our study. The same G variant in a heterozygous state might be associated with the non-hyperandrogenic phenotype of PCOS. The presence of *CYP21A2* genetic variants IVS2-13A/C (rs6467, c.293-13A/C), rs6453 (c.293-44G>T), rs6451 (c.293-67C>A/G), rs369651496 (c.293-104del), and rs6474 (c.308G>A/p.R103L) has been related to earlier menarche, increased DHEAS levels, and variations in blood pressure and lipid levels. Further studies on *CYP21A2* pathogenic variants and polymorphisms modulating adrenal steroidogenesis in different ethnic groups might help explain the PCOS phenotype heterogeneity and variable metabolic risks, which is a prerequisite for developing more specific therapeutic approaches.

## Figures and Tables

**Table 1:** Anthropometric and laboratory characteristics of the investigated PCOS women (n = 60). BMI—body mass index; HOMA-IR—homeostatic model assessment for insulin resistance; HDL-cholesterol—high-density lipoprotein cholesterol; LDL-cholesterol—low-density lipoprotein cholesterol; DHEAS—dehydroepiandrosterone sulfate; LH—luteinizing hormone; FSH—follicle-stimulating hormone; Min.—minimal value; and Max.—maximal value.

Parameter	Median/(%)	Interquartile Range	Min.–Max.
Age (years)	23.50	21.00–26.00	18.00–45.00
BMI (kg/m^2^)	27.67	22.85–35.09	17.17–52.89
Acne (%)	43.3		
Hirsutism (%)	70.0		
Menarche (years)	13.00	11.87–13.00	9.00–17.00
Irregular menstruation (%)	88.3		
Live births (%)	10.0		
Systolic blood pressure (mmHg)	110.00	105.00–120.00	90.00–140.00
Diastolic blood pressure (mmHg)	70.00	70.00–80.00	60.00–90.00
HOMA-IR	3.45	2.02–4.74	0.65–30.46
Glucose (mmol/L) 0 min	5.32	5.01–5.65	4.16–8.00
Glucose (mmol/L) 120 min	6.06	5.00–7.40	3.18–9.72
Insulin (µIU/mL) 0 min	14.40	8.59–19.54	3.50–85.67
Insulin (µIU/mL) 120 min	51.91	34.26–106.42	5.58–253.40
Cholesterol-total (mmol/L)	4.53	4.12–5.04	2.62–6.77
HDL-cholesterol (mmol/L)	1.28	1.06–1.56	0.78–2.35
LDL-cholesterol (mmol/L)	2.95	2.55–3.68	0.70–5.47
Triglycerides (mmol/L)	0.95	0.67–1.17	0.31–2.94
Testosterone (nmol/L)	1.61	1.26–1.88	0.09–3.08
Androstenedione (nmol/L)	14.10	10.72–19.12	4.60–31.40
17-OH-progesterone (nmol/L)	4.10	3.07–5.52	0.37–12.60
DHEAS (µmol/L)	9.31	7.16–13.22	3.14–19.79
LH (IU/L)	7.54	4.39–11.31	1.50–34.95
FSH (IU/L)	5.67	4.80–7.30	2.80–9.60
Prolactin (µIU/mL)	287.00	201.50–407.00	73.200–883.00

**Table 2:** Genotypes and allele frequency observed among investigated PCOS patients in the present study.

Genetic Variant	Common Homozygote		Heterozygote		Rare Homozygote		*p*		q	
	Geno- Type	N	Geno- Type	N	Geno- Type	N	Major Allele	Frequency	Minor Allele	Frequency
rs6467 (IVS2-13A/C, c.293-13A/C)	**AA**	**24**	**СА**	**25**	**СС**	**10**	**A**	**0.62**	**C**	**0.38**
rs6474 (c.308G>A)	**GG**	**18**	**GA**	**36**	**AA**	**6**	**G**	**0.60**	**A**	**0.40**
rs6453 (c.293-44G>T)	**GG**	**52**	**GT**	**5**	**TT**	**1**	**G**	**0.94**	**T**	**0.06**
rs6451 (c.293-67C>A/G)	**CC**	**39**	**CA** **CG** **GA**	**11** **5** **3**	**AA** **GG**	**1** **1**	**C**	**0.78**	**A** **G**	**0.13** **0.08**
rs369651496 (c.293-104del)	**7G7G**	**35**	**7G/6G**	**18**	**6G6G**	**7**	**7G**	**0.73**	**6G**	**0.27**

**Table 3:** Clinical and laboratory characteristics of women with polycystic ovarian syndrome according to rs6467 polymorphism (the IVS2-13A/C>G G allele carrier has been excluded from the analyses). BMI—body mass index; HOMA-IR—homeostatic model assessment for insulin resistance; HDL-cholesterol—high-density lipoprotein cholesterol; LDL-cholesterol—low-density lipoprotein cholesterol; DHEAS—dehydroepiandrosterone sulfate; LH—luteinizing hormone; FSH—follicle-stimulating hormone; and IQR—interquartile range.

Parameter	rs6467 C/C and C/A Genotypes (n = 35)		rs6467 A/A Genotype (n = 24)		*p*
	Median/%	IQR	Median/%	IQR	
Age (years)	23.00	21.00–26.00	25.00	22.00–28.00	*0.361*
BMI (kg/m^2^)	27.96	22.81–36.66	27.67	23.88–33.79	*0.763*
Acne (%)	48.60		37.50		*0.436*
Hirsutism (%)	74.30		66.70		*0.569*
Menarche (years)	13.00	12.00–13.00	13.000	11.00–13.00	*0.777*
Irregular menstruation (%)	88.6		87.5		*1.000*
Live births (%)	2.9		20.8		***0.036***
Systolic blood pressure (mmHg)	110.00	110.00–120.00	110.00	100.00–120.00	*0.169*
Diastolic blood pressure (mmHg)	75.00	70.00–80.00	70.00	67.50–77.50	*0.054*
HOMA-IR	4.08	2.06–5.67	3.09	2.04–4.23	*0.331*
Glucose (mmol/L) 0 min.	5.45	5.04–5.81	5.13	5.01–5.57	*0.115*
Glucose (mmol/L) 120 min.	6.31	5.38–7.56	5.63	4.68–6.78	*0.112*
Insulin (µIU/mL) 0 min.	16.80	8.54–22.68	12.94	9.10–17.31	*0.323*
Insulin (µIU/mL) 120 min.	87.28	39.79–120.42	38.77	30.29–65.01	***0.042***
Cholesterol-total (mmol/L)	4.61	4.24–5.15	4.51	4.03–5.02	*0.589*
HDL-cholesterol (mmol/L)	1.28	1.03–1.49	1.29	1.18–1.65	*0.316*
LDL-cholesterol (mmol/L)	2.98	2.64–3.74	2.82	2.42–3.68	*0.502*
Triglycerides (mmol/L)	0.96	0.70–1.16	0.82	0.64–1.18	*0.463*
Testosterone (nmol/L)	1.53	1.26–1.83	1.63	1.32–2.00	*0.683*
17-OH-progesterone (nmol/L)	3.95	3.00–5.50	4.20	3.05–6.35	*0.721*
Androstenedione (nmol/L)	14.20	11.85–18.57	12.90	9.60–21.57	*0.714*
DHEAS (µmol/L)	9.38	7.05–13.22	9.14	7.54–13.20	*0.867*
LH (IU/L)	7.39	4.25–11.00	7.95	5.15–11.76	*0.439*
FSH (IU/L)	5.80	5.10–7.42	5.30	4.50–6.90	*0.385*
Prolactin (µIU/mL)	284.50	194.00–400.00	324.50	208.50–424.00	*0.444*

**Table 4:** Clinical and laboratory characteristics of women with polycystic ovarian syndrome according to rs6474 (c.308 G>A, p.Arg103Lys) polymorphism. BMI—body mass index; HOMA-IR—homeostatic model assessment for insulin resistance; HDL-cholesterol—high-density lipoprotein cholesterol; LDL-cholesterol—low-density lipoprotein cholesterol; DHEAS—dehydroepiandrosterone sulfate; LH—luteinizing hormone; FSH—follicle-stimulating hormone; and IQR—interquartile range.

Parameter	rs6474 G/G Genotype (n = 18)		rs6474 G/A and A/A Genotypes (n = 42)		*p*
	Median/%	IQR	Median/%	IQR	
Age (years)	22.50	21.00–25.00	24.00	21.00–26.00	*0.481*
BMI (kg/m^2^)	26.66	21.79–32.69	28.04	23.38–36.68	*0.239*
Acne (%)	27.80		50.00		*0.157*
Hirsutism (%)	55.60		76.20		*0.132*
Menarche (years)	12.50	11.000 to 13.000	13.00	12.00–13.00	*0.296*
Irregular menstruation (%)	83.30		90.50		*0.418*
Live births (%)	11.10		9.50		*1.000*
Systolic blood pressure (mmHg)	110.00	100.00–110.00	112.50	110.00–120.00	***0.019***
Diastolic blood pressure (mmHg)	70.00	70.00–80.00	75.00	70.00–80.00	*0.195*
HOMA-IR	3.21	2.07–4.60	3.63	1.99–5.97	*0.594*
Glucose (mmol/L) 0 min.	5.26	5.00–5.55	5.32	5.02–5.81	*0.302*
Glucose (mmol/L) 120 min.	7.32	5.22–7.51	5.88	4.76–7.19	*0.281*
Insulin (µIU/mL) 0 min.	13.85	9.08–18.36	14.88	7.19–23.71	*0.796*
Insulin (µIU/mL) 120 min.	55.46	33.63–119.32	50.67	35.19–106.75	*0.682*
Cholesterol-total (mmol/L)	4.46	4.13–5.38	4.53	4.12–5.04	*0.859*
HDL-cholesterol (mmol/L)	1.29	1.01–1.46	1.27	1.08–1.62	*0.936*
LDL-cholesterol (mmol/L)	3.06	2.56–3.82	2.86	2.54–3.61	*0.611*
Triglycerides (mmol/L)	0.76	0.55–1.07	0.97	0.69–1.19	*0.135*
Testosterone (nmol/L)	1.46	0.61–1.80	1.64	1.34–2.03	*0.125*
17-OH-progesterone (nmol/L)	4.20	3.05–5.30	4.10	3.05–5.92	*0.779*
Androstenedione (nmol/L)	10.75	8.65–14.35	15.80	12.67–20.62	***0.022***
DHEAS (µmol/L)	8.38	4.90–12.97	9.34	7.55–13.37	*0.307*
LH (IU/L)	6.88	5.62–8.34	7.78	4.30–11.68	*0.566*
FSH (IU/L)	5.60	4.80–7.40	5.67	4.70–7.20	*0.909*
Prolactin (µIU/mL)	277.00	162.00–423.00	287.00	213.75–401.00	*0.553*

**Table 5:** Clinical and laboratory characteristics of women with polycystic ovarian syndrome according to rs6453 (c.293-44G>T) polymorphism. BMI—body mass index; HOMA-IR—homeostatic model assessment for insulin resistance; HDL-cholesterol—high-density lipoprotein cholesterol; LDL-cholesterol—low-density lipoprotein cholesterol; DHEAS—dehydroepiandrosterone sulfate; LH—luteinizing hormone; FSH—follicle-stimulating hormone; and IQR—interquartile range.

Parameter	rs6453 G/G Genotype (n = 54)		rs6453 G/T and T/T Genotypes (n = 6)		*p*
	Median/%	IQR	Median/%	IQR	
Age (years)	23.00	21.00–26.00	24.50	23.00–26.00	*0.766*
BMI (kg/m^2^)	27.87	23.38–36.59	23.23	19.48–27.96	*0.055*
Acne (%)	42.60		50.00		*1.000*
Hirsutism (%)	72.20		50.00		*0.352*
Menarche (years)	13.00	12.00–13.00	11.50	11.00–13.00	*0.148*
Irregular menstruation (%)	90.70		66.70		*0.140*
Live births (%)	11.10		0.00		*1.000*
Systolic blood pressure (mmHg)	110.00	110.00–120.00	110.00	100.00–120.00	*0.593*
Diastolic blood pressure (mmHg)	70.00	70.00–80.00	75.00	70.00–80.00	*0.372*
HOMA-IR	3.75	1.99–5.36	2.59	2.06–3.43	*0.430*
Glucose (mmol/L) 0 min.	5.32	5.00–5.66	5.32	5.14–5.55	*0.941*
Glucose (mmol/L) 120 min.	5.98	4.83–7.40	7.32	6.01–7.61	*0.297*
Insulin (µIU/mL) 0 min.	15.02	8.09–20.39	10.32	9.40–15.03	*0.521*
Insulin (µIU/mL) 120 min.	49.11	32.98–105.20	105.80	63.53–107.67	*0.317*
Cholesterol-total (mmol/L)	4.53	4.13–5.05	4.47	4.02–4.75	*0.498*
HDL-cholesterol (mmol/L)	1.25	1.00–1.62	1.45	1.29–1.48	*0.460*
LDL-cholesterol (mmol/L)	2.95	2.56–3.76	2.89	2.24–3.30	*0.538*
Triglycerides (mmol/L)	0.95	0.68–1.19	0.65	0.55–0.96	*0.109*
Testosterone (nmol/L)	1.60	1.26–2.03	1.64	1.53–1.68	*0.980*
17-OH-progesterone (nmol/L)	4.10	2.92–5.57	4.00	3.60–4.90	*0.613*
Androstenedione (nmol/L)	14.05	10.75–19.75	17.50	10.45–18.10	*0.916*
DHEAS (µmol/L)	9.14	6.92–11.43	15.18	13.59–17.35	***0.003***
LH (IU/L)	7.97	4.95–11.59	5.38	4.35–6.59	*0.084*
FSH (IU/L)	5.67	5.00–7.50	5.40	4.100–6.60	*0.382*
Prolactin (µIU/mL)	287.00	203.75–409.00	257.50	162.00–404.00	*0.437*

**Table 6:** Clinical and laboratory characteristics of women with polycystic ovarian syndrome according to rs6451 (c.293-67C>A/G) polymorphism. BMI—body mass index; HOMA-IR—homeostatic model assessment for insulin resistance; HDL-cholesterol—high-density lipoprotein cholesterol; LDL-cholesterol—low-density lipoprotein cholesterol; DHEAS—dehydroepiandrosterone sulfate; LH—luteinizing hormone; FSH—follicle-stimulating hormone; and IQR—interquartile range.

Parameter	rs6451 C/C Genotype (n = 39)		rs6451 Other Genotypes (n = 21)		*p*
	Median/%	IQR	Median/%	IQR	
Age (years)	23.00	21.00–28.50	24.00	21.75–25.00	*0.680*
BMI (kg/m^2^)	28.37	23.50–36.66	26.56	21.06–32.04	*0.137*
Acne (%)	35.90		57.10		*0.172*
Hirsutism (%)	69.20		71.40		*1.000*
Menarche (years)	13.00	12.00–13.25	12.00	11.00–13.00	***0.007***
Irregular menstruation (%)	92.30		81.00		*0.226*
Live births (%)	10.30		9.50		*1.000*
Systolic blood pressure (mmHg)	120.00	110.00–120.00	110.00	100.00–110.00	***0.023***
Diastolic blood pressure (mmHg)	70.00	70.00–80.00	70.00	68.75–80.00	0.485
HOMA-IR	3.80	1.72–5.67	3.25	2.24–4.28	0.969
Glucose (mmol/L) 0 min.	5.34	5.00–5.79	5.30	5.03–5.57	0.411
Glucose (mmol/L) 120 min.	5.88	4.76–7.41	6.12	5.09–7.38	0.761
Insulin (µIU/mL) 0 min.	15.31	7.11–22.88	13.63	9.37–18.41	0.883
Insulin (µIU/mL) 120 min.	61.30	38.23–110.65	38.77	30.29–82.79	0.197
Cholesterol-total (mmol/L)	4.61	4.12–5.18	4.44	4.16–4.80	0.476
HDL-cholesterol (mmol/L)	1.24	1.04–1.63	1.33	1.20–1.46	0.325
LDL-cholesterol (mmol/L)	2.97	2.55–3.72	2.82	2.48–3.43	0.733
Triglycerides (mmol/L)	0.95	0.70–1.19	0.70	0.59–1.10	0.204
Testosterone (nmol/L)	1.64	1.27–1.91	1.59	1.20–1.83	0.636
17-OH-progesterone (nmol/L)	4.40	3.10–5.60	3.70	2.87–4.75	0.528
Androstenedione (nmol/L)	14.20	11.15–19.02	12.85	9.95–20.85	0.772
DHEAS (µmol/L)	9.11	7.16–12.53	10.64	7.34–14.10	0.370
LH (IU/L)	8.94	5.65–11.88	6.66	4.25–8.15	0.056
FSH (IU/L)	5.75	5.05–7.55	5.35	4.70–6.65	0.185
Prolactin (µIU/mL)	262.50	204.00–393.00	352.00	192.00–415.50	0.367

**Table 7:** Clinical and laboratory characteristics of women with polycystic ovarian syndrome according to rs369651496 (c.293-104del) polymorphism. BMI—body mass index; HOMA-IR—homeostatic model assessment for insulin resistance; HDL-cholesterol—high-density lipoprotein cholesterol; LDL-cholesterol—low-density lipoprotein cholesterol; DHEAS—dehydroepiandrosterone sulfate; LH—luteinizing hormone; FSH—follicle-stimulating hormone; and IQR—interquartile range.

Parameter	rs369651496 7G/7G Genotype (n = 35)		7G/6G and 6G/6G Genotypes (n = 25)		*p*
	Median/%	IQR	Median/%	IQR	
Age (years)	24.00	22.00–26.00	23.00	21.00–26.25	*0.302*
BMI (kg/m^2^)	27.00	21.97–34.97	28.00	23.92–35.31	*0.481*
Acne (%)	48.60		36.00		*0.430*
Hirsutism (%)	62.90		80.00		*0.253*
Menarche (years)	12.50	11.00–13.00	13.00	12.00–13.75	*0.211*
Irregular menstruation (%)	91.40		84.00		*0.436*
Live births (%)	17.10		0.00		***0.036***
Systolic blood pressure (mmHg)	110.00	100.00–120.00	110.00	110.00–120.00	*0.148*
Diastolic blood pressure (mmHg)	70.00	70.00–80.00	70.00	70.00–80.00	*0.994*
HOMA-IR	2.89	1.96–4.78	3.86	2.25–4.83	*0.584*
Glucose (mmol/L) 0 min.	5.31	5.03–5.71	5.34	5.00–5.62	*0.887*
Glucose (mmol/L) 120 min.	6.06	5.17–7.32	5.90	4.69–7.44	*0.826*
Insulin (µIU/mL) 0 min.	11.85	8.54–20.16	16.26	9.15–19.22	*0.524*
Insulin (µIU/mL) 120 min.	42.39	35.37–105.65	68.00	32.98–108.30	*0.576*
Cholesterol-total (mmol/L)	4.73	4.24–5.54	4.32	4.05–4.78	***0.033***
HDL-cholesterol (mmol/L)	1.31	1.18–1.59	1.25	0.99–1.48	*0.083*
LDL-cholesterol (mmol/L)	3.20	2.63–4.08	2.73	2.44–3.19	***0.042***
Triglycerides (mmol/L)	0.82	0.62–1.16	1.06	0.76–1.17	*0.384*
Testosterone (nmol/L)	1.48	1.26–1.77	1.72	1.32–2.09	*0.165*
17-OH-progesterone (nmol/L)	3.70	3.07–5.50	4.50	3.00–5.75	*0.673*
Androstenedione (nmol/L)	12.80	10.67–18.00	15.85	14.10–23.40	*0.224*
DHEAS (µmol/L)	9.56	7.36–14.62	9.27	6.84–11.35	*0.480*
LH (IU/L)	7.87	4.36–11.11	7.47	4.60–11.80	*0.905*
FSH (IU/L)	5.70	4.77–7.42	5.65	4.85–6.85	*0.868*
Prolactin (µIU/mL)	295.50	201.00–404.00	287.00	203.25–412.25	*0.848*

## Data Availability

Data are available from the corresponding author after reasonable request and permission from the local authorities.
